# Dynamics of quantum correlation between separated nitrogen-vacancy centers embedded in plasmonic waveguide

**DOI:** 10.1038/srep15513

**Published:** 2015-10-23

**Authors:** Wan-li Yang, Jun-Hong An, Cheng-jie Zhang, Chang-yong Chen, C. H. Oh

**Affiliations:** 1 State Key Laboratory of Magnetic Resonance and Atomic and Molecular Physics, Wuhan Institute of Physics and Mathematics, Chinese Academy of Sciences, Wuhan 430071, China; 2 Center for Interdisciplinary Studies & Key Laboratory for Magnetism and Magnetic Materials of the MoE, Lanzhou University, Lanzhou 730000, China; 3 Centre for Quantum Technologies, National University of Singapore, Singapore 117543, Singapore; 4 Department of Physics, Shaoguan University, Shaoguan, Guangdong 512005, China

## Abstract

We investigate the dynamics of quantum correlation between two separated nitrogen vacancy centers (NVCs) placed near a one-dimensional plasmonic waveguide. As a common medium of the radiation field of NVCs propagating, the plasmonic waveguide can dynamically induce quantum correlation between the two NVCs. It is interesting to find that such dynamically induced quantum correlation can be preserved in the long-time steady state by locally applying individual driving on the two NVCs. In particular, we also show that a large degree of quantum correlation can be established by this scheme even when the distance between the NVCs is much larger than their operating wavelength. This feature may open new perspectives for devising active decoherence-immune solid-state optical devices and long-distance NVC-based quantum networks in the context of plasmonic quantum electrodynamics.

The plasmonic quantum electrodynamics (QED) has emerged as an attractive route towards scalable solid-state systems for trapping various optical emitters^1^,^2^,^3^,^4^,^5^,^6^,^7^,^8^,^9^. Parallel to the cavity QED^10^, plasmonic QED has become another popular platform and provided new opportunities for studying and controlling the basic light-matter interaction. Confining the electromagnetic field in the regions well below the diffraction limit^11^, the plasmonic modes could manipulate light via the localized surface plasmons in photonics. Therefore, plasmons give rise to very strong local fields around emitters and can be guided along the interface in the form of a traveling wave known as a surface plasmon-polariton (SPP)^12^. Additionally, the well-developed fabrication techniques make the plasmonic nanostructure a promising candidate for quantum control, quantum optics, and quantum information processing (QIP), e.g., single photons sources^13^, atomic spectroscopy^14^, focusing^15^, lasing^16^, superradiance^17^, and single-plasmon emission^18^.

In the parallel development, integrated dipole emitters featuring surface plasmons are showing remarkable characteristics and novel phenomena, where ultrasmall optical mode volume in plasmonic nanostructures offers predominant conditions for reaching emitter-plasmons strong coupling regime. Recent experimental progresses on the hybrid emitter-plasmons system have provided obvious evidence for strong coupling between molecules and surface plasmons via a splitting of the surface-plasmon mode dispersion^19^, and strong coupling between quantum dots and surface plasmons via the vacuum Rabi splitting^20^, and strong coupling of the emission from a single NVC to the channel plasmon polaritons supported by a V-groove plasmonic waveguide^21^, respectively. On the other hand, much effort has also been devoted to theoretically address the emitter-plasmons coherent coupling^22^,^23^,^24^,^25^, and entanglement between separated emitters mediated by plasmonic modes^4^,^26^,^27^.

Our objective in this paper is to establish stable quantum correlation between two separated NVCs embedding in a one-dimensional (1D) plasmonic waveguide (PW). It is expected that our study not only provides information of how quantum correlation evolves in time, but also suggests efficient ways toward practical purposes with quantum correlation. We are interested in two questions: How to develop efficient methods for tailoring the steady-state quantum correlation, and to what extent quantum correlation generation and dynamics in such a hybrid system can be efficiently controlled by adjusting the key tunable parameters of the system? Meanwhile, it is significant to protect quantum correlation from the ubiquitous decoherence in quantum world^28^,^29^. Therefore, the evolution of the quantum correlation for our NVC system in the PW environment is certainly of great interest, and it is vital to develop efficient way to overcome the detrimental influence of the decoherence from the PW environment. The experimental observation of peculiar features for quantum correlation and its great extension in a solid-state system with genuine noise renders the use of quantum correlation as a physical resource in QIP more practicable^28^,^29^.

Combining the unique properties of plasmonic modes in waveguide with the attractive features of NVC (robust room-temperature spin coherence^30^ and efficient optical addressability, control, and readout^31^,^32^,^33^) makes this coupled-NVC-PW model an ideal hybrid system for applications ranging from QIP to quantum computation. Our work is based on recent experimental and theoretical progresses, e.g., the realization of efficient coupling of a NVC to propagating plasmonic modes by measuring the enhanced spontaneous emission decay rates^34^,^35^. The broadband enhancement of spontaneous emission enabled by nanoplasmonic approaches offers the possibility of strong coupling to NVCs, which was otherwise difficult to achieve by conventional quantum optical techniques^36^. The remarkable features of our model include: the two different types of PW-induced interactions between the NVCs, such as *g*_12_ (the coherent dipole-dipole coupling rate) and Γ_12_ (the incoherent coupling rate), which influence the quantum correlation dynamics of NVCs in different ways, and they can effectively be switched on/off by changing the distance between the emitters. In addition, we find that a finite steady-state quantum correlation can be established by individually applying the external driving on the NVCs separated with long distance, which is quantitatively different from the result of approaching zero in the absence of continuous driving. Such stable quantum correlation generation between distant NVCs is the preprequisites for realizing large-scale NVC-based quantum networks^37^,^38^,^39^.

## Results

### System and model

**Figure 1:**
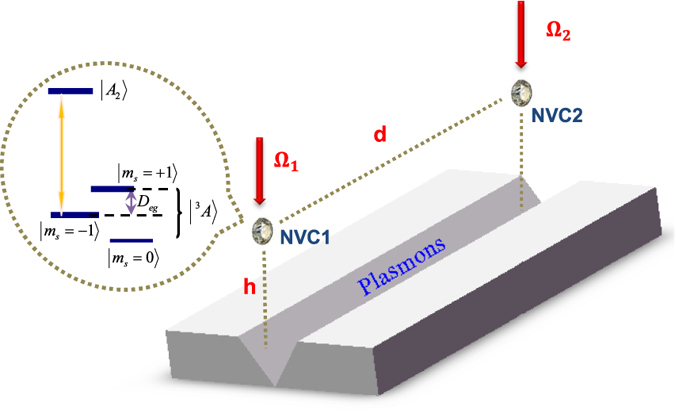
The composite NVC-PW system consists of a 1D plasmonic waveguide and two identical NVCs in diamond nanocrystals, where the two red arrows denote the external laser driving on the NVCs. The inset shows the level structure of a NVC, where the electronic ground state 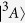 is a spin triplet state, and 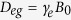 is the level splitting induced by an external magnetic field *B*_0_ with *γ*_*e*_ the electron gyromagnetic ratio. The orange arrow denotes the coupling between NVC and PW.

We consider two separated NVCs (NVC1 and NVC2) coupled to the modes supported by a 1D PW, as shown in Fig. 1. Each NVC is negatively charged with two unpaired electrons located near the vacancy, usually treated as electron spin-1. The PW modes with *σ*^+^ polarization are coupled to the transition from the ground state sublevels 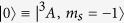 to one of the excited states 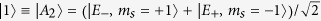
40,41,42,43 with the transition frequency *ω*_0_. Tracing out the degrees of freedom of the PW and employing the Born-Markovian approximation, the master equation for two NVCs can be obtained^4^,^5^


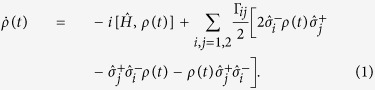


The Hamiltonian in Eq. (1) is given by


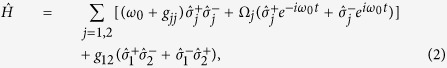


where Ω_*j*_ is the Rabi frequency of the resonant laser driving on the *j*-th NVC with the raising and lowering operators 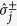. It is interesting to see from Eq. (1) that the PW, as a common medium to confine the radiation field of the two NVCs, can not only induce individual spontaneous emission (with rate Γ_*jj*_) and frequency shift *g*_*jj*_ to each NVCs, but also induce correlated spontaneous emission (with rate Γ_12_ = Γ_21_) and coherent dipole-dipole interaction *g*_12_ between the two NVCs by the exchange of virtual plasmons. *g*_*ij*_ and Γ_*ij*_ are determined by


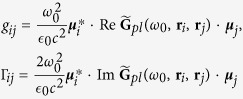


where 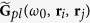 is the plasmon contribution of Green’s function with regards to the dipole moments ***μ***_*i*_ and ***μ***_*j*_ locating at the position **r**_*i*_ and **r**_*j*_, respectively. One can find that both *g*_*ij*_ and Γ_*ij*_ can be extracted from the knowledge of the dipole moments and the classical Green’s tensor in the presence of the PW. The dipole moment can be inferred from the measurement of the decay rate of one NVC in vacuum, whose Green’s tensor is well known^5^. We will label 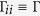 as the spontaneous emission rates of the individual NVC under the condition that the two identical NVCs are placed at two equivalent positions along the PW. At optical frequencies, for NVC-PW distances larger than about 10 *nm*, the frequency shift *g*_*jj*_ is very small and will be neglected in the following.

It is worth mentioning that the coherent and incoherent contributions of the PW to the coupling between the NVCs are proportional to the real and imaginary parts of the Green’s function, respectively. Under the condition that the NVC-NVC interaction is predominantly plasmon-assisted, they can be reduced to


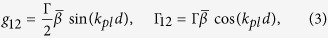


where 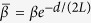 with *d* being the interqubit distance, *k*_*pl*_ = 2*π*/*λ*_*pl*_ (λ_*pl*_ = 637 nm in our case) and *L* is the wave-number and propagation length of the plasmon, respectively. The *β* factor is a parameter that measures the fraction of the emitted radiation captured by the propagating mode. It can be close to 1 due to the subwavelength nature of the plasmonic modes^5^. An interesting feature shown in Eqs. (3) is the feasibility of modulating the phase difference between the plasmon-mediated coherent and incoherent parts of the coupling via tailoring the distance *d*. This might allow the switching on/off one of the two contributions and offer the opportunity for controlling the degree of quantum correlation between NVCs.

In this work we focus on the dynamical evolution of the quantum correlation. We will characterize the quantum correlation by both quantum discord (QD)^44^,^45^,^46^,^47^,^48^ and entanglement of formation (EoF)^49^, respectively. As one of the well known measures of quantum correlations, quantum entanglement characterized by EoF plays essential roles in quantum system and quantum information science. However, it cannot exhaust the nonclassicality in the correlations^28^. It is believed that QD characterizes the quantumness of correlations more generally than entanglement. It has been shown that QD plays a resource role in more and more protocols in QIP^28^,^29^,^50^.

For simplicity, our analysis is restricted to the case of two NVCs prepared in the Bell-like states 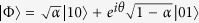. In the following we will first study the case of no external driving, and then apply the results obtained to the case of external driving on the NVCs.

### Dynamics of quantum correlation

#### No external continuous driving

From Eqs. (1, 2) we can see that the PW has dual actions on the decoherence dynamics of the NVCs. On one hand, the PW acts as a decoherence source on each individual NVC, which is believed to be destructive to quantum correlation between the two NVCs. On the other hand, it acts as a mediation party to induce coherent (*g*_12_) and incoherent (Γ_12_) interactions, which is expected to be constructive in establishing quantum correlation between the NVCs. Therefore, the dynamics of quantum correlation reflects the intricate balance and competition between the two contributions. As a result, the overall dynamics may exhibit some complex competition between the (coherent or incoherent) interaction induced oscillation and the decoherence induced damping behaviors.

Note that there is *π*/2 phase difference between *g*_12_ and Γ_12_, which allows switching off one of the two contributions (*g*_12_ and Γ_12_) while maximizing the other by just choosing the interqubit distance^5^. In what follows, we calculate the exact dynamics of correlations by directly solving the master equation (1) for the initial state 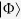. The obtained time-dependent density matrix is 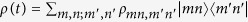, where the non-zero elements are


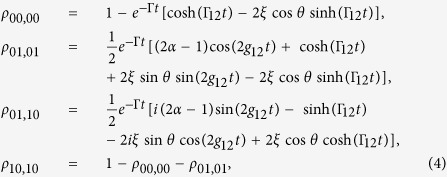


with 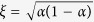. Once the density matrix *ρ*(*t*) is obtained, the quantum correlations of the two NVCs can be quantified by means of the EoF and QD, respectively. Here the EoF is quantified by concurrence^51^ as 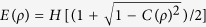 with 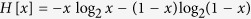. The concurrence in our case takes the form 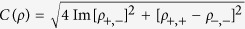 with 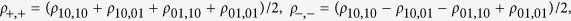 and 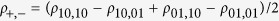. Although much effort has been made for the X states (i.e., states with the nonzero elements of the density matrix lying only along the diagonal or antidiagonal in the product basis), there is still no analytical expression of QD for general two-qubit states. The main obstacle comes from the complicated optimization procedure to the measurement basis^52^. Therefore, our investigation resorts to the numerical simulation.

**Figure 2:**
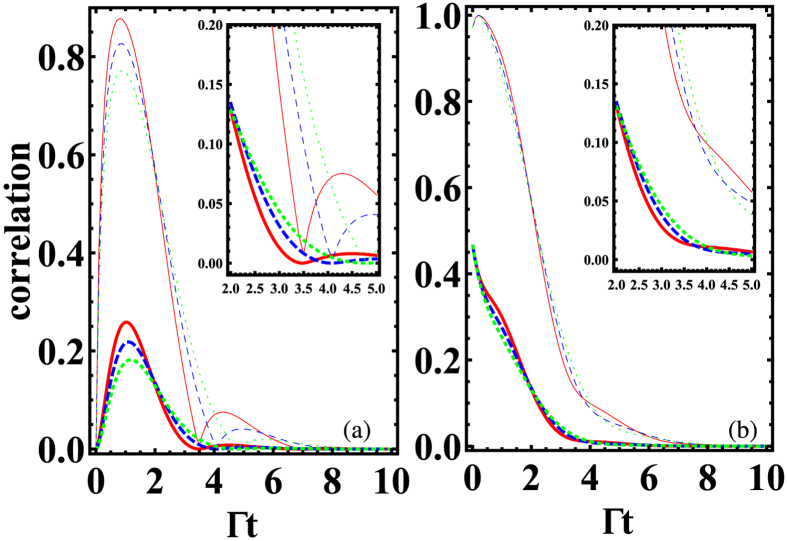
Time evolution of the quantum correlations calculated by QD (thick line) and EoF (thin line) for different initial state 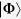 in the absence of driving. (**a**) *α* = 0 and *θ* = 2*π* (**b**) *α* = 0.1 and *θ* = 2*π*. The solid, dashed, and dotted lines denote the case of 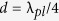, 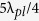, and 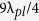, respectively. The parameters *L* = 2 *μm* and *β* = 0.94 are used.

**Figure 3:**
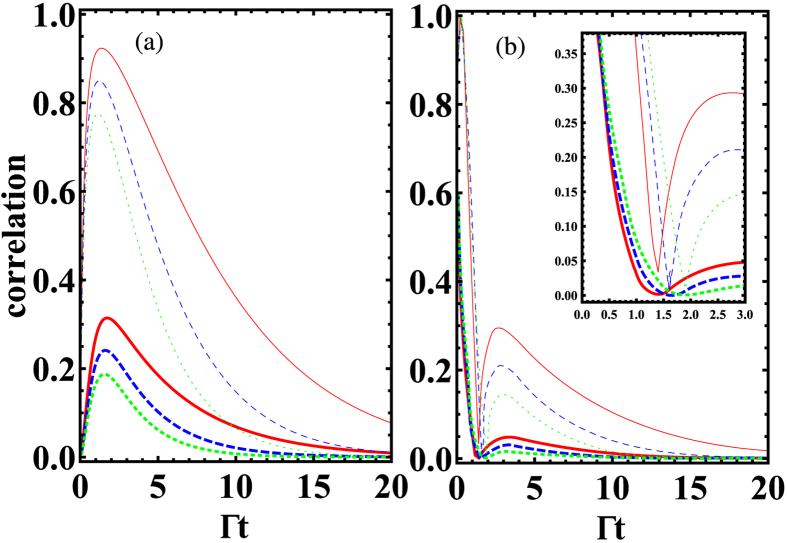
Time evolution of the quantum correlations calculated by QD (thick line) and EoF (thin line) for different initial state 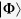 in the absence of driving. (**a**) *α* = 0 and *θ* = 2*π*. (**b**) *α* = 0.2 and *θ* = 2*π*. The solid, dashed, and dotted lines denote the case of 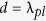, 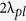, and 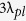, respectively. The parameters *L* = 2 *μm* and *β* = 0.94 are used.

Figures 2 and 3 present the quantum correlation dynamics of the NVCs with different initial states. We consider two different sets of interqubit distances *d* corresponding to two typical cases: i.e., the large dipole-dipole coupling case (Fig. 2) and the large dissipation case (Fig. 3).

In the former case 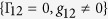, when the NVCs are initially prepared in a uncorrelated state 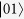, as shown in Fig. 2a, the quantum correlations calculated by EoF display an obvious revival then disappear gradually in the long-time limit. This revival feature is unapparent for QD (see the subgraph of Fig. 2a). Here the coherent energy exchange between the two NVCs (due to *g*_12_) leads to the generation of and revival behavior of quantum correlations. In contrast to this feature, if the NVCs are initially prepared in a state with initial correlation, as shown in Fig. 2b, the quantum correlations EoF and QD will exhibit nonexponential decays to zero due to the information ceaselessly losing into the environment, which results from the spontaneous emission (with rate Γ_*jj*_) of the individual NVC. In the latter case 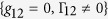, both EoF and QD between NVC1 and NVC2 vanish at a relatively slower speed (as shown in Fig. 3) than the ones in the former case. Although no dipole-dipole interactions exist in this case, the transient quantum correlations still can be established due to the incoherent interaction from the correlated spontaneous emission (with rate Γ_12_) between the NVCs. Another difference from the former case is that the EoF and QD behave similarly with obvious revival phenomena when the NVCs are initially prepared in a state with initial correlation (*α* = 0.2), as shown in Fig. 3b.

All the above-mentioned difference between these two cases indicates that the unique features of PW play a vital role in the quantum correlation evolution of the NVCs confined in the plasmonic modes. Note that the same qualitative tendency is observed for QD and EoF during the time evolution with EoF being always larger than QD for both cases. From Eqs. (3) it is clear that a variation of distances *d* produces a change in the oscillations of the density matrix elements and therefore in the correlations dynamics: the degree of quantum correlations is suppressed because the two contributions (*g*_12_ and Γ_12_) is decreased, which is also a common feature shown in Figs 2 and 3.

#### Under external continuous driving

We now study the case where the NVCs are pumped by two external resonant lasers. A remarkable character in this driven-dissipation process is that a finite quantum correlations, which are labeled by Eof(∞) and QD(∞), can be obtained in the long-time limit. It implies that the transient quantum correlation dynamically induced by the common PW can be stabilized to its long-time steady state by locally applying individual driving fields on the two NVCs and adjusting the key parameters. Such a scheme, which does not resort to the direct interaction between the NVCs to establish quantum correlation, might be employed as an important resource for devising active decoherence-immune solid-state optical devices.

**Figure 4:**
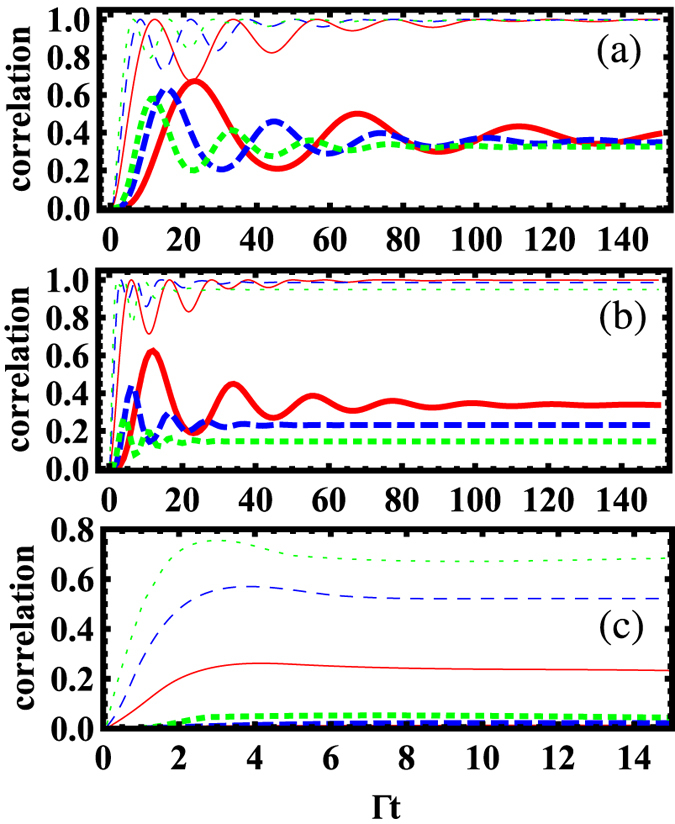
Time evolution of the quantum correlations calculated by QD (thick line) and EoF (thin line) under three different laser configurations. (**a**) The solid, dashed, and dotted lines denote the case of 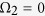 and 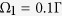, 0.15Γ, and 0.2Γ, respectively. (**b**) The solid, dashed, and dotted lines denote the case of 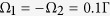, 0.2Γ, and 0.3Γ, respectively. (**c**) The solid, dashed, and dotted lines denote the case of 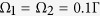, 0.2Γ, and 0.3Γ, respectively. The parameters *d* = λ_*pl*_ and 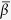 = 0.99 are used.

To get a clear picture on how the quantum correlation evolves to its long-time value, we plot in Fig. 4 the time evolution of EoF and QD under three different driving cases: (*i*) only one NVC is pumped (i.e. 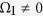 and 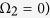, (*ii*) both NVCs are pumped with the opposite phases (i.e. 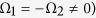, and (*iii*) both NVCs are pumped with the identical phases (i.e. 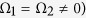. One can find that the case (*iii*) has the shortest time scale for reaching the steady state, while the case (*i*) has the longest time scale. Furthermore, a series of fast oscillation behavior can be found in the cases (*i*) and (*ii*), while it is absent in the case (*iii*). Such oscillation arises from the competition between the different physical processes induced by the two external driving fields with different magnitudes and phases. However, once the driving fields are at the identical magnitudes and phases, the oscillation disappears [see Fig. 4(c)]. Therefore, the external lasers allow an additional degree of freedom of quantum control to quantum correlation. Compared the driving-on case with the driving-off case, we can find that the continuous driving can make the quantum correlation induced by the incoherent interaction terms Γ_12_ and the coherent interaction terms *g*_12_ stabilized to its long-time steady state.

**Figure 5:**
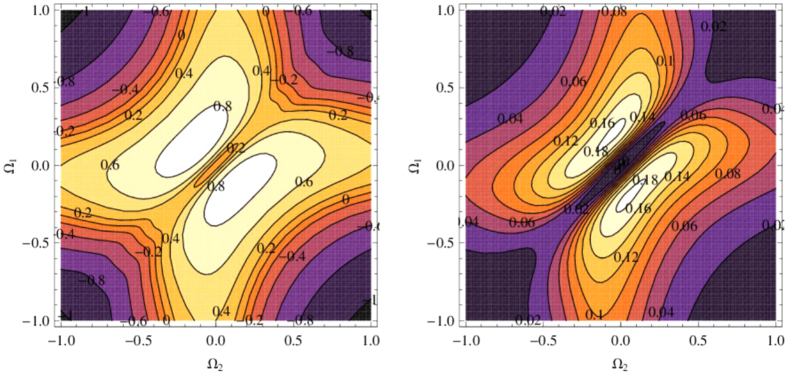
The steady-state quantum correlations Eof(∞) and QD(∞) versus the parameters Ω_1_ and Ω_2_. The parameters *L* = 2 *μm*, *d* = λ_*pl*_, and *β* = 0.94 are used.

Another interesting feature is that the obtained stable quantum correlations are not a monotonic function of the magnitudes of driving lasers. We plot in Fig. 5 the dependence of Eof(∞) and QD(∞) on the magnitudes of the lasers, i.e. the Rabi frequencies Ω_1_ and Ω_2_. Here an optimal driving condition on the magnitudes and phases of the two driving lasers to achieve the maximal quantum correlations can be obtained. Therefore, our method offers an efficient way to control both the time-dependent dynamics and the steady-state quantum correlation between NVCs, which enhances the potential of NVC-PW systems as a candidate for scalable solid-state QIP, and provides a useful information to practical experiment for generating long-distance quantum correlation between NVCs.

**Figure 6:**
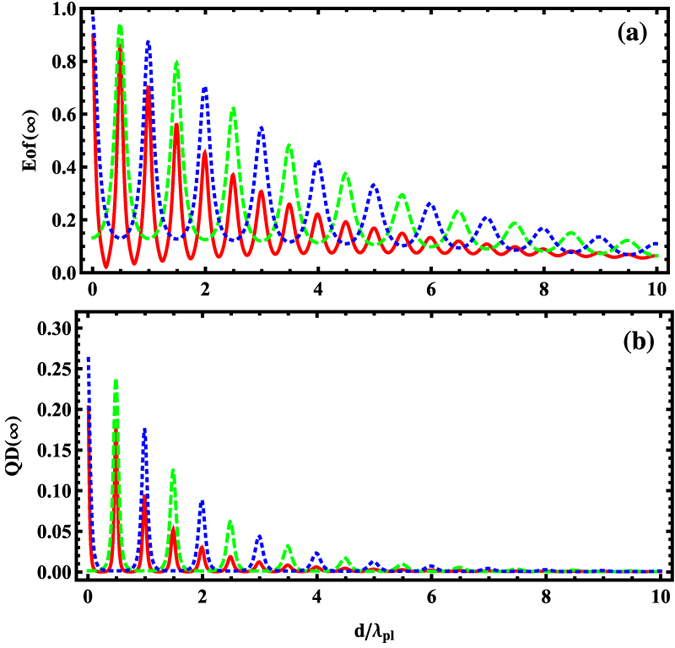
The quantum correlations Eof(∞) and QD(∞) versus the interqubit distance *d*, where the solid, dotted, and dashed lines denote the case of {Ω_1_ = 0.1Γ, Ω2 = 0}, {Ω1 = −Ω2 = 0.1Γ}, and {Ω1 = Ω2 = 0.1Γ, respectively. The parameters Γ = 1, *L* = 2 *μm*, and *β* = 0.94 are used.

Figure 6 displays the steady-state quantum correlations as a function of the interqubit distance *d* under the above cases (*i*–*iii*). It is observed that both Eof(∞) and QD(∞) exhibit a periodic oscillation behavior in period λ_*pl*_ with a decreasing amplitude of oscillation as the growth of the distance between NVCs. Furthermore, for sufficiently separated NVCs with distances much larger than the operating wavelength λ_*pl*_, a large amount of quantum correlations in steady state can also be achieved. The cases (*ii*) and (*iii*) always perform better in generating quantum correlations than the case (*i*), where only one laser driving is applied on one NVC. Figure 6 also tells us that one could get a tunable quantum correlation between NVCs by adjusting the interqubit distance *d*. It could be used for long-distance quantum communication in realistic experiments, which is one of the critical goals in quantum information science.

## Discussion

We survey the relevant parameters and experimental feasibility. In our case we consider realistic values *β* = 0.94, *L* = 2 *μm*, and the vertical distance *h* = 180 *nm*, which correspond to a transition wavelength of λ_*pl*_^4^. Besides, the previous work shows that the strong NVC-PW coupling regime is accessible within current technology when working at very low temperatures 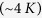, and highly enhanced spontaneous emission with Purcell factors over 1000 at room temperature for NVCs through further optimization^5^. Confining the light field to small effective volumes (far below the diffraction limit) in plasmonic modes enables stronger coupling to the optical emitter, despite having low quality factors owing to Ohmic losses. Meantime, the strategies about reducing the damping of the material or incorporate cavities into plasmonic structures to increase the *Q*-factor of the plasmonic modes, have been pursued to make the strong-coupling regime be entered more easily^1^.

In experiments, quantum correlations quantified by QD have been investigated as important physical resources in solid-state systems with a real noisy environment^28^,^29^, ion trap^53^, optical system^54^, liquid NMR systems^55^, and so on. Here the QD can be measured by the state tomography technique^56^. For example, the measurement in different spin bases can be achieved through observing both the in-phase and quadrature components of the electron spin echo. Additionally, compared all the amplitudes of the Rabi nutations in the tomography procession to the amplitude of electron spin Rabi nutation, the experimental density matrix of qubits can be fully reconstructed^28^,^29^. Furthermore, in our two-NVC system, quantum correlation could also be detectable by measuring cross terms of a second-order coherence function, which can be realized in a Hanbury Brown-Twiss-like experiment by detecting photon-photon correlations of the emission from the NVCs^5^.

In summary, we have studied the dissipative dynamics of quantum correlation between a pair of NVCs placed near a 1D PW. We have revealed that a dynamical quantum correlation can be generated due to the coherent dipole-dipole interaction and the incoherent correlated spontaneous emission induced by the exchange of virtual plasmons of the PW, but decays to zero asymptotically due to the individual spontaneous emission induced by the PW. However, the dynamically generated quantum correlation can be stabilized to the steady state by applying two local driving lasers on the NVCs. Our results furnish helpful suggestion on the future design of more complex plasmonic structures for quantum control. Our study highlights the benefits associated with building nano-photonic systems that use surface plasmons in the quantum regime.

## Methods

We will introduce the definition of quantum correlation by both QD^44^,^45^ and EoF^49^, respectively. The EoF is quantified by concurrence^51^ as 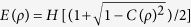 with 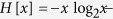 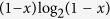. The concurrence is defined as 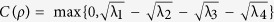, where the decreasing-order-arranged quantities λ_*i*_ are the eigenvalues of the matrix 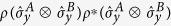 with 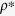 the complex conjugation of *ρ* and 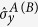 the Pauli matrix acting on the subsystem *A*(*B*).

QD is defined as the minimum difference between two ways on defining mutual information (MI), 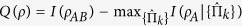 with 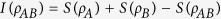 the quantum MI and 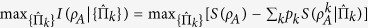 the maximal MI when a measurement is performed on subsystem *B*^43^. Here 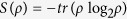 is the von Neumann entropy of density matrix *ρ*, 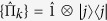, 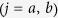 is a completely positive operator valued measure on the subsystem *B*, where 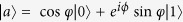 and 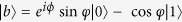, and the projectors 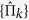 satisfy the relation 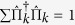, and 
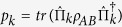
 is the respective probabilities.

## Additional Information

**How to cite this article**: Yang, W.-l. *et al*. Dynamics of quantum correlation between separated nitrogen-vacancy centers embedded in plasmonic waveguide. *Sci. Rep*. **5**, 15513; doi: 10.1038/srep15513 (2015).
